# Remote Hind-Limb Ischemia Mechanism of Preserved Ejection Fraction During Heart Failure

**DOI:** 10.3389/fphys.2021.745328

**Published:** 2021-11-11

**Authors:** Rubens P. Homme, Yuting Zheng, Irina Smolenkova, Mahavir Singh, Suresh C. Tyagi

**Affiliations:** Department of Physiology, University of Louisville School of Medicine, Louisville, KY, United States

**Keywords:** congestive heart failure, creatine kinase isoforms, viral myocarditis, matrix metalloproteinases, tissue remodeling

## Abstract

During acute heart failure (HF), remote ischemic conditioning (RIC) has proven to be beneficial; however, it is currently unclear whether it also extends benefits from chronic congestive, cardiopulmonary heart failure (CHF). Previous studies from our laboratory have shown three phases describing CHF viz. (1) HF with preserved ejection fraction (HFpEF), (2) HF with reduced EF (HFrEF), and (3) HF with reversed EF. Although reciprocal organ interaction, ablation of sympathetic, and calcium signaling genes are associated with HFpEF to HFrEF, the mechanism is unclear. The HFrEF ensues, in part, due to reduced angiogenesis, coronary reserve, and leakage of endocardial endothelial (EE) and finally breakdown of the blood-heart barrier (BHB) integrity. In fact, our hypothesis states that a change in phenotype from compensatory HFpEF to decompensatory HFrEF is determined by a potential decrease in regenerative, proangiogenic factors along with a concomitant increase in epigenetic memory, inflammation that combinedly causes oxidative, and proteolytic stress response. To test this hypothesis, we created CHF by aorta-vena-cava (AV) fistula in a group of mice that were subsequently treated with that of hind-limb RIC. HFpEF vs. HFrEF transition was determined by serial/longitudinal echo measurements. Results revealed an increase in skeletal muscle musclin contents, bone-marrow (CD71), and sympathetic activation (β2-AR) by RIC. We also observed a decrease in vascular density and attenuation of EE-BHB function due to a corresponding increase in the activity of MMP-2, vascular endothelial growth factor (VEGF), caspase, and calpain. This decrease was successfully mitigated by RIC-released skeletal muscle exosomes that contain musclin, the myokine along with bone marrow, and sympathetic activation. In short, based on proteome (omics) analysis, ∼20 proteins that appear to be involved in signaling pathways responsible for the synthesis, contraction, and relaxation of cardiac muscle were found to be the dominant features. Thus, our results support that the CHF phenotype causes dysfunction of cardiac metabolism, its contraction, and relaxation. Interestingly, RIC was able to mitigate many of the deleterious changes, as revealed by our multi-omics findings.

## Introduction

**FIGURE 1:**
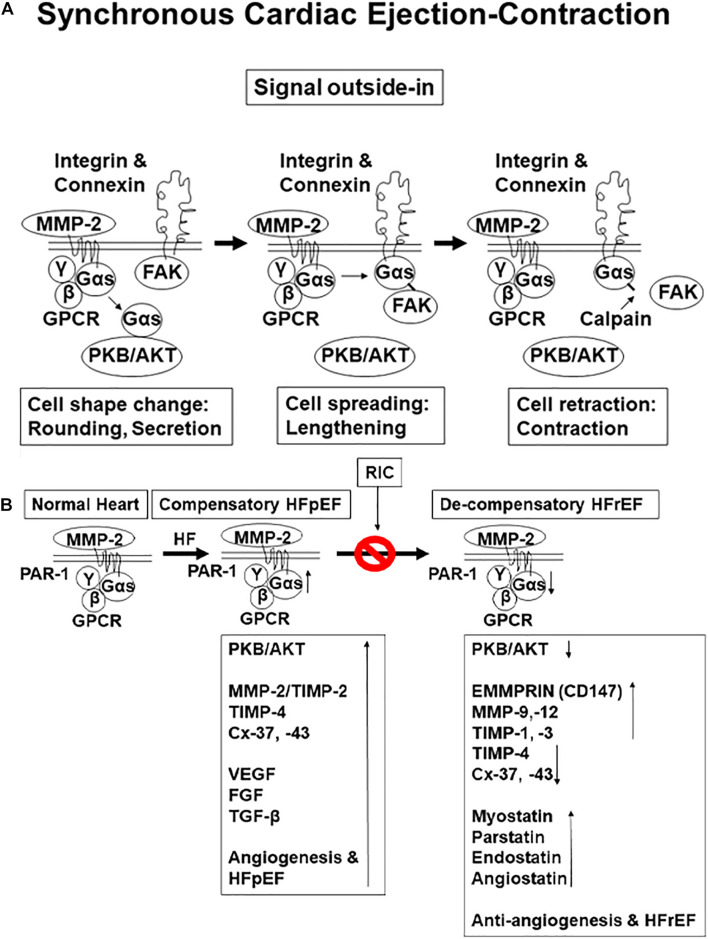
**(A)** Physiological function of a contractile cell: synthesis, relaxation, and contraction (i.e., cell protein synthesis, spreading, and migration). **(B)** The hypothesis is that with aging chronic volume overload by preload-induced heart failure (HF) initially is with preserved ejection fraction (HFpEF); however, persistence increases in load lead to HF with reduced ejection fraction. This is caused by the decrease in perfusion and coronary reserve and reduced angiogenesis as compared to preserved ejection fraction (HFrEF).

Previous studies from our laboratory have demonstrated that cardiac muscle physiology is associated with cell metabolism, cell migration, cell spreading, and cell contraction that are analogous to contraction and relaxation of the cardiac cycle (Tyagi, 1997a,b; Tummalapalli and Tyagi, 1999). Since matrix metalloproteinase 2 (MMP-2) is constitutively expressed, it helps release growth factors from extra cellular matrix (ECM) during normal remodeling (i.e., during development and angiogenesis) (Tyagi, 1997a,b; Tummalapalli and Tyagi, 1999). The signal outside-in is instigated by integrins, connexins, MMP-2, protease-activated receptor-1 (PAR1), G protein-coupled receptor (GPCR), and as we knew that G protein alpha s subunit (Gαs) is linked to protein kinase B (PKB) (AKT) that causes the cell metabolic synthesis (Azevedo et al., 2001; Cox et al., 2002; Ciccarelli et al., 2021). Then, subsequently, Gαs links to focal adhesion kinase (FAK) and causes cell spreading and relaxation. This linkage is broken by calpain that results in cell contraction (Figure 1A). Although many studies have established the protective role of remote ischemic conditioning (RIC) in ischemic dysfunction of the left ventricle (LV) (Crimi et al., 2013; Donato et al., 2013; Heusch et al., 2015; White et al., 2015; Haller et al., 2017; Kim et al., 2017; Majumder et al., 2019), the mechanism is unclear. The studies employing the forearm-cuff procedure were found to be controversial since they did not produce ischemia. Interestingly, recent studies revealed mitigation of the size post-RIC of the infarct in the cohort of patients (Crimi et al., 2013; Heusch et al., 2015; White et al., 2015; Haller et al., 2017; Kim et al., 2017). Although RIC mitigated cardiopulmonary heart failure (CHF), the mechanism is unclear. Studies have shown the role of sympathetic and other stimuli from muscle during cardioprotection (Givvimani et al., 2012; Donato et al., 2013; Majumder et al., 2019). Previous studies from our laboratory have demonstrated an increase in the levels of antiangiogenic endostatin and angiostatin during decompensatory heart failure (HF) (Givvimani et al., 2010, 2012). The hypothesis of this study is that RIC releases exosomes from skeletal muscle containing inciting molecules such as musclin, the myokine, and that mitigates the CHF (Figure 1B). The contracting myofibrils release musclin peptides, which confer beneficial effects on muscle (Subbotina et al., 2015). Thus, cardiomyocytes-derived extracellular vesicles (EVs) injected into the infarcted hearts have been shown to promote ejection-fraction recovery leading to functional recovery; however, the role of musclin and exosomes released during RIC on myocardium is unknown (Liu et al., 2018). Therefore, the premises of this study are that the hind-limb RIC produces full ischemia to the leg and in that process releases exosomes, muscle hormones such as musclin, and that provides systemic, as well as cardiac muscle protection (Subbotina et al., 2015).

## Materials and Methods

### Animal Protocol and Treatment

Male and female 8–12 weeks old mice, wild type (WT) (C57BL/6J), were purchased from the Jackson Laboratory (Bar Harbor, ME, United States). The animal procedures were reviewed and subsequently approved by the Institutional Animal Care and Use Committee (IACUC) of the University of Louisville. Furthermore, the animal care and guidelines of the National Institutes of Health (NIH, United States) were also adhered to. Since aging causes chronic congestive CHF primarily due to a chronic increase in preload volume, we created aorta-vena-cava fistula (AVF) below the kidney in WT mice treated with or without remote hind-limb ischemia (RIC) for a total of 16 weeks. AVF was created in anesthetized WT mice between the aorta and the caudal vena cava, ∼0.5 cm below the kidneys, using a 30-gauge needle. An infrarenal AVF creates an unambiguous model of chronic volume overload. In this model, arterial blood, below the kidneys, rapidly enters the venous circulation and overloads the ventricles without the contribution of the stimulation of circulating factors (Givvimani et al., 2010). This model omits the effects of mechanical injuries, such as mitral valve regurgitation and vasodilatation (Tsoporis and Leenen, 1988; Turcani and Jacob, 1998). We selected C57BL/6J mice since this strain serves as the accepted background for genetically engineered mice procedures. Also, results obtained by others have suggested extensive genetic homology between mice and humans (Qureshi et al., 1999). Since CHF due to AVF causes right ventricle (RV), pulmonary, and LV dysfunction, we analyzed RV, LV, and the lung. Due to the chances of oxidative stress and proteolytic activation happening concurrently and thus, to avoid an early activation of MMP and to prevent AVF-mediated oxidative and proteolytic stresses subsequently, we created RIC 1 day after AVF surgical process. To create RIC, the left femoral artery was ligated at the same time as the AVF for 16 weeks. The ligation was established by measuring the decreased blood flow with the help of laser Doppler system monitoring. The counter lateral leg served as the control limb for the experiments (Majumder et al., 2018a,b, 2019). A minimum of 5–8 animals/group were used in the experiments.

### Gravimetric and Creatine Kinase Isoform Measurements

Body weight, lung weight, and heart weight were measured at the time of sacrifice. The blood levels of creatine kinase (CK) activity were also measured. In brief, the tissue-specific injury was determined by measuring the CK isoforms in the serum samples from each group of mice. The CK-MM represents the cardiac- and skeletal-muscle-specific isoform, while the CK-BB is primarily a nerve-specific and kidney-specific isoform, respectively. From each mouse, 10 μl of plasma was mixed with 1 μl of activator and loaded onto the CK gel as instructed by the manufacturer (QuickGel^®^ CK Vis Isoenzyme Procedure; Helena Laboratories, TX, United States). The gels were run at 400 V for 4:15 min. The standard (ST) amounts of CK isoforms were also loaded in parallel to the samples (Miller et al., 2000; Stanisic et al., 2021). Blood exosomes were isolated as described earlier (Familtseva et al., 2019).

### Western Blotting

Antibodies and reagent antibodies for disheveled associated activator of morphogenesis 1 (DAAM 1), DAAM 2, and regulated in development DNA damage response 1 (REDD 1) were purchased from Santa Cruz Biotechnology (Santa Cruz, CA, United States), dynamin-related protein 1 (Drp1), DNA methyl transferase (DNMT) 3B the mitochondrial transcription factor A (TFAM) from Abcam (Cambridge, MA, United States), phosphatidylethanolamine methyltransferase (PEMT) from Lifespan Biosciences, Seattle, WA, United States, and the glyceraldehyde-3-phosphate dehydrogenase (GAPDH) was purchased from Millipore Sigma, St. Louis, MO, USA. After post 48-h treatment period, cardiac tissue protein was isolated using protein extraction buffer [radio-immuno precipitation assay (RIPA) lysis buffer, protease inhibitor cocktail, and phenylmethylsulphonyl fluoride (PMSF)]. Lysates were spun in an extraction buffer for 12 h and then centrifuged at 12,000 × *g* for 15 min. The supernatant was transferred to new tubes, and protein concentrations were analyzed *via* Bradford protein estimation assay. Samples were run on a 10/12% sodium dodecyl sulfate (SDS)-polyacrylamide gel with Tris-glycine SDS buffer. The gel was transferred electrophoretically overnight onto a polyvinylidene difluoride (PVDF) membrane at 4°C. The membrane was blocked with a 5% milk solution for 1 h. Primary antibodies were diluted at a concentration of 1:1,000 in Tris buffered saline with Tween-20 (TBST) and incubated on the membrane overnight. All membranes were washed in TBST solution four times and then incubated with secondary horseradish peroxidase (HRP) conjugated antibody solution for 1 h at room temperature. Four TBST washing steps followed before membranes were developed using a chemiluminescent substrate in a BioRad ChemiDoc (Hercules, CA, United States). Band intensity was determined using densitometry analysis. Beta-actin was used to normalize protein loading. Equal amounts of total protein (50 μg) were resolved on SDS-polyacrylamide gel electrophoresis (PAGE) and transferred to polyvinylidene membranes. The membranes were probed overnight at 4°C with primary antibodies followed by 2 h incubation in secondary antibodies. The signal capturing was performed using the Bio-Rad ChemiDoc XRS + system and Image Lab software (Bio-Rad, Hercules, CA, United States). The relative optical density of protein bands was analyzed using gel software Image Lab 3.0. The membranes were stripped and re-probed with GAPDH as a loading control.

### Cardiac Ring Preparation and Endothelial Myocyte Coupling

The determination of endothelial function in an isolated papillary muscle preparation does not demonstrate what happens in the entire transmyocardial wall. Therefore, to determine endocardial endothelial (EE) function, acetylcholine was perfused in a Langendorff preparation (Wang and Morgan, 1992; Gattuso et al., 1999; Mujumdar and Tyagi, 1999; Cox et al., 2002). However, this also does not differentiate the specific contribution of the regional ischemia, hypertrophy, stunning, and/or hibernation of myocytes in the myocardial wall. Rather it gives a global contractile response to cardiotonic agents. Furthermore, it does not separate the effects of the LV from the RV. Thus, using cardiac ring preparation, we compared data obtained from cardiac rings with the data obtained by Langendorff preparation in hypertensive rats and found similar results. In addition, the cardiac ring preparation separates the effect of the LV from RV. To determine the specific regional differences in contractile function, the rings will be prepared to include or to exclude the homogenous or non-homogeneous regions of the transmural myocardial wall. The endothelial-dependent cardiomyocyte function will be measured in cardiac rings prepared from the LV and RV separately from intact and endothelial-denuded hearts from sham and AVF mice. The response to acetylcholine (an endothelial-dependent) and nitroprusside (endothelial-independent) relaxation will be measured.

### Barium Contrast X-Ray Angiography

To determine vascular density, barium contrast X-angiography was performed as described earlier. In brief, after pentobarbital anesthesia, mice were infused with barium sulfate (0.1 g/ml) in 50 mmol/L Tris-buffer (pH 5.0) at a constant flow (∼1 ml/min) and pressure with a syringe pump through the common carotid artery. Heparin (20 U/ml) was used along with barium sulfate to visualize the vessels. The angiograms were captured using the Carestream whole animal X-ray imaging system (Carestream Molecular Imaging, Woodbridge, CT, United States) as previously described, and the vessel density was quantified using VesSeg tool (Institute for Signal Processing, University of Luebeck, Lübeck, Germany) (Machens et al., 2006; Givvimani et al., 2011; Majumder et al., 2018b;George et al., 2019).

### Echocardiography and Cardiac Function

Echocardiography was used to determine overall myocardial function using the ultrasound with the Vevo 2100 imaging system. The cardiac and aortic data were collected as described previously. Experimental animals were placed supine on a warm platform (37°C) under isoflurane anesthesia and fixed. Using an MS550D (22–25 MHz) transducer, the thoracic cavity was imaged. Aortic arch velocity and cardiographic function were assessed in pulse wave and color Doppler modes. The transducer probe was placed on the left hemithorax of the mice in the partial left decubitus position. Two-dimensionally targeted M-mode echocardiograms were obtained from a short-axis view of the LV at or just below the tip of the mitral valve leaflet and were recorded. LV size and the thickness of the LV wall were also measured. Only the M-mode ECHO with well-defined continuous interfaces of the septum and posterior wall were collected (George et al., 2020; Singh et al., 2020).

### Statistical Analysis

Data from sham, AVF, sham + RIC, and AVF + RIC were analyzed statistically using the one-way ANOVA to know the difference between the groups, including a Tukey’s *post hoc* analysis for comparison of groups, comparing AVF with sham and AVF + RIC with AVF groups. Each group has enough mice for the experiments, and the data were presented as the mean ± SEM (*n* = 5–8 animals/group), *^∗^p* < 0.05.

## Results

### Physiological Function, Aging, Morphological Changes, Tissue Injury, Exosomes, and Cardiac Signaling

**FIGURE 2:**
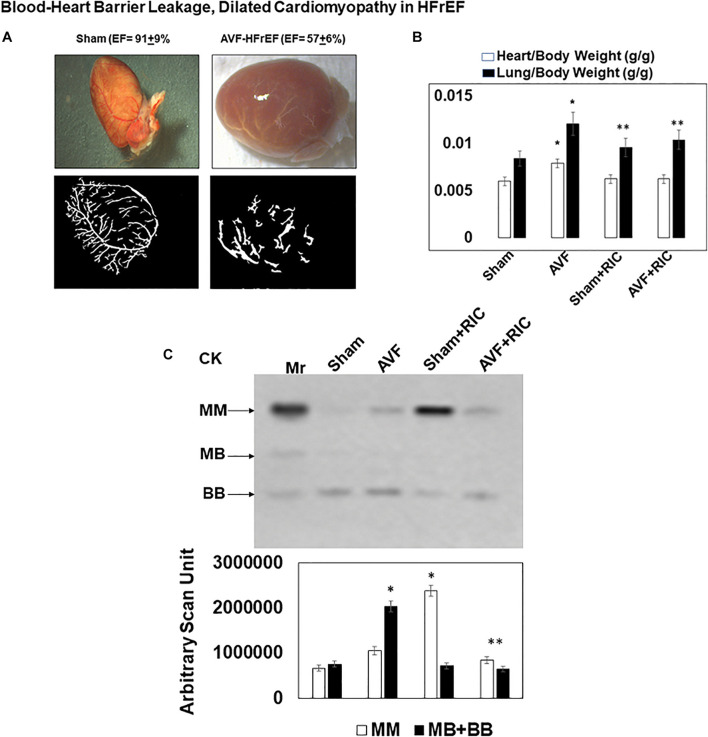
**(A)** Morphology of a sham and cardiopulmonary heart failure (CHF)-AVF heart: the shape of the heart normally is a spindle shape; however, in CHF, it takes a global shape. The vascular density is decreased in CHF, and the vessels are more dilated and leakier as compared to the normal heart. This suggested disruption of blood-heart barrier (BHB) and dilated cardiomyopathy. **(B)** The heart and lung weight of sham and AVF mice treated with and without the hind-limb remote ischemic condition (RIC). The heart-lung weights were increased in CHF indicative of congestive HF. Bar graphs with ±SD are representative of *n* = 5–8. **(C)** The in-gel creatine kinase (CK) activity (upper panel). Both the skeletal muscle and cardiac and neuronal CKs were increased in AVF and RIC. Interestingly the RIC mitigated the cardiac damage. Bar graphs of relative scan units with ±SD are representative of *n* = 5–8. * and ** indicate significant difference (*p* < 0.05) either to “sham without RIC” or to “0” week.

**FIGURE 3:**
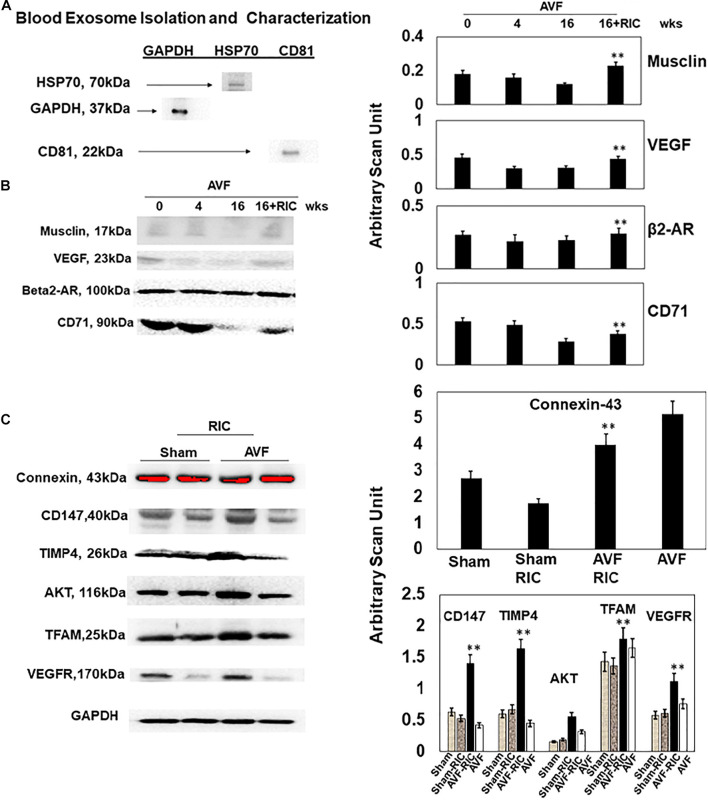
**(A)** Identification and characterization of exosomes isolated from mouse hearts. Western blot analysis using exosome markers (HSP70, CD81, and GAPDH). **(B)** Representative Western blot analysis. The blood exosome levels of musclin, VEGF, beta2-AR, and CD71 (a marker of bone marrow stem cell). Bar graph representation of with + SD from *n* = 5–8 (right panel). **(C)** The representative Western blot analysis of signaling cardiac levels of connexin-43, EMMPRIN (CD147), TIMP-4, AKT, TFAM, and VEGFR. Right panel: Bar graph representation with ±SD from *n* = 5–8. ** indicates significant difference (*p* < 0.05) either to “0” week or to “sham without RIC.”

**FIGURE 4:**
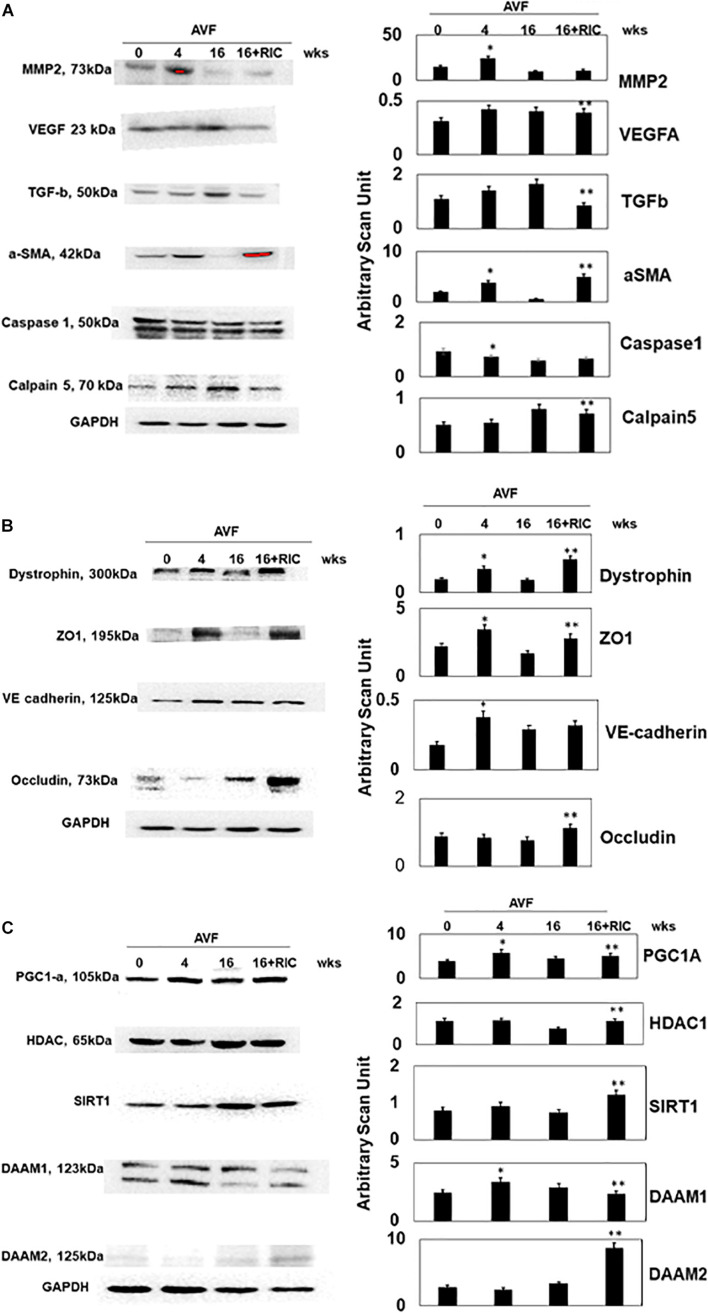
**(A)** The Western blot analysis of inciting cardiac levels of MMP2, VEGF, TGF-b, a-SMA, caspase1, and calpain5. Bar graph of relative scan unit representation with ±SD from *n* = 5–8. **(B)** The Western blot analysis of intercellular connecting cardiac levels of dystrophin, ZO1, VE cadherin, and occludin. Bar graph of relative scan unit representation with ±SD from *n* = 5–8. **(C)** The Western blot analysis of regenerative cardiac levels of PGC1-a, HDAC, SIRT1, DAAM 1, and DAAM 2. Bar graph of relative scan unit representation with ±SD from *n* = 5–8. * and ** indicate significant difference (*p* < 0.05) either to “sham without RIC” or to “0” week.

The levels of vascular density were decreased in HFrEF. However, these vessels were bigger in size than controls, indicative of leakage of the vessels and causing dilated cardiomyopathy. This also suggested the breakdown of blood-heart barrier (BHB) function/integrity (Figure 2A). The heart and lung weights were increased in AVF-HF, suggestive of congestive HF. Interestingly, this HF was mitigated by RIC (Figure 2B). The cardiac and skeletal muscle injuries were increased by both AVF and RIC. Interestingly, this injury was mitigated together in RIC with AVF (Figure 2C). We isolated and characterized the exosomes released during RIC (Figure 3A). The exosomes isolated from RIC groups revealed elevated levels of musclin, Beta 2-AR, CD71, and vascular endothelial growth factor (VEGF). These results suggested stimulation of bone-marrow, sympathetic activation, and skeletal muscle myokines by RIC (Figure 3B; Marsee et al., 2010; Kalani et al., 2014, 2016). The signal outside-in axis was activated by RIC in terms of inducing connexin-43, CD147, tissue Inhibitor of metalloproteinase-4 (Timp-4), AKT, TFAM, and VEGF (Figure 3C) along with the remodeling axis as by influence by TGF-b, VEGFR, and alpha-smooth muscle actin (a marker of myofibroblast), intracellular caspase1, and calpain by RIC in CHF-AVF hearts (Figure 4A). The cell-cell connexin and adhesive junctions were stimulated by RIC during CHF (Figure 4B). The regeneration axis was stimulated by RIC in CHF by increasing PGC1-a, HDAC1, SIRT1, DAAM 1, and DAAM 2 (differential expression) (Figure 4C).

### Epigenetics, Cellular Stress, and Mitigation by Remote Ischemic Conditioning

**FIGURE 5:**
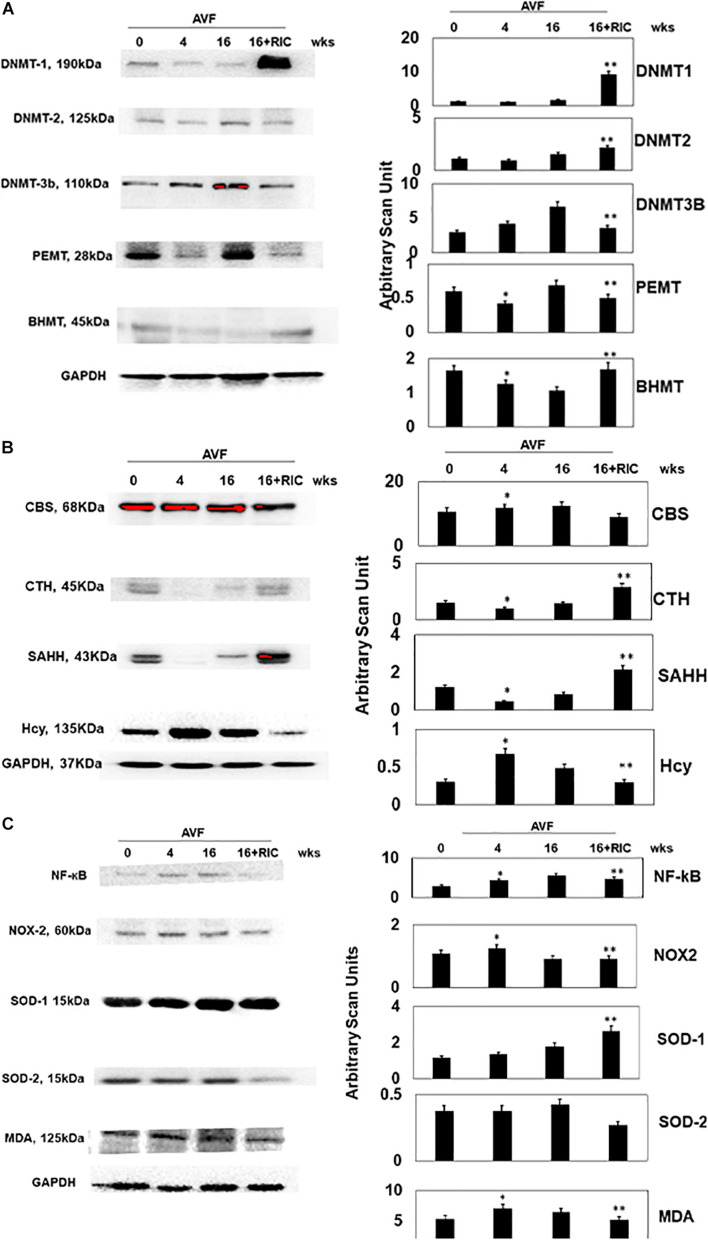
**(A)** The Western blot analysis of epigenetic cardiac levels of DNA methyl transferase 1 (DNMT1), DNMT2, DNMT3b, phosphatidylethanolamine methyltransferase (PEMT), and BHMT. Bar graph of relative scan unit representation with ±SD from *n* = 5–8. **(B)** The Western blot analysis of homocysteine metabolism cardiac levels of CBS, CTH, S-adenosine homocysteine hydrolase (SAHH), and homocysteine (Hcy). Bar graph of relative scan unit representation with ±SD from *n* = 5–8. **(C)** The Western blot analysis of inflammatory and oxidative stress pathway cardiac levels of NF-kB, NOX2, SOD1, SOD2, and malonaldehyde (MDA). Bar graph of relative scan unit representation with ±SD from *n* = 5–8. * and ** indicate significant difference (*p* < 0.05) either to “sham without RIC” or to “0” week.

The epigenetic cascades were instigated in CHF by RIC, as indicated by the levels of DNMT-1, 2, and 3b, PEMT, and betaine–homocysteine S-methyltransferase (BHMT) (Figure 5A). Since epigenetics is the hallmark of homocysteine (Hcy) generation, we measured the Hcy metabolic axis. The results suggested that RIC increased CTH and S-adenosine homocysteine hydrolase (SAHH) and decreased Hcy in CHF (Figure 5B). Since high Hcy induces oxidative stress, we measured the NF-kB, NOx2, SOD-2, SOD2, and malonaldehyde (MDA, a marker of oxidative stress). The results suggested a decrease in oxidative stress after RIC treatment during CHF (Figure 5C).

### Cardiac Function Assessment, Cardiac Remodeling, and Its Mitigation by Myokine Derived From Skeletal Muscle Exosomes

**FIGURE 6:**
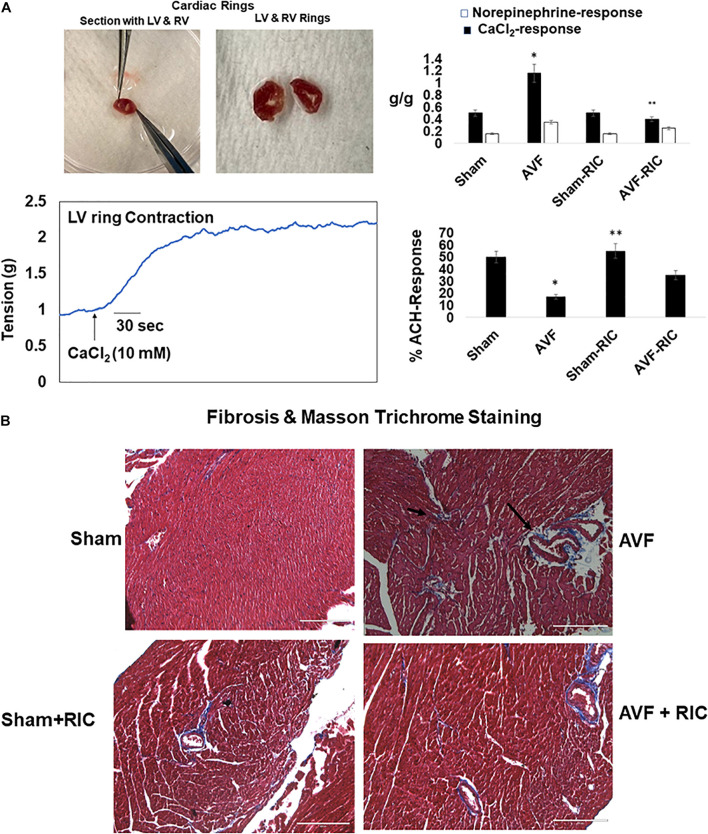
**(A)** Representative *ex vivo* cardiac contractile (CaCl_2_ and norepinephrine) (left panels) and endothelial-dependent (acetylcholine) function in cardiac left ventricle (LV) ring preparation (bottom right panel). Bar graph of relative scan unit representation with ±SD from *n* = 5–8. **(B)** The representative trichrome blue collagen fibrosis stain in sham and AVF hearts with and without RIC. * and ** indicate significant difference (*p* < 0.05) either to “sham without RIC” or to “0” week.

**FIGURE 7:**
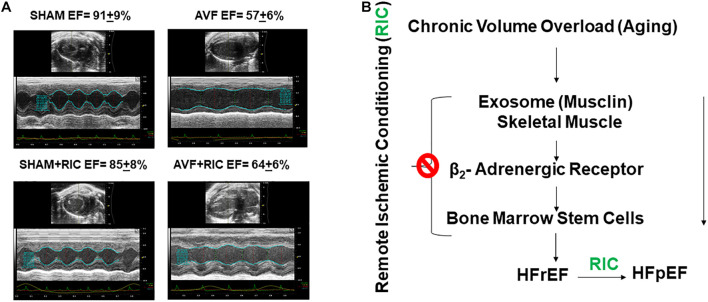
**(A)** The representative echocardiography and ejection fraction (EF) of sham and AVF hearts from mice treated with and without RIC. **(B)** HFrEF is caused by decreases in skeletal muscle myokine and RIC stimulates the exosome from skeletal muscle and mitigates CHF.

The cardiac contraction to CaCl_2_ and norepinephrine were attenuated in AVF and was mitigated by RIC. The acetylcholine response and endothelial-dependent cardiac muscle relaxation were abrogated by AVF volume overload. Interestingly, the RIC mitigated this endothelial dysfunction (Figure 6A). The cardiac interstitial and perivascular fibrosis were robust in AVF hearts; however, the fibrosis was attenuated by RIC (Figure 6B). The echocardiography data revealed that AVF chronic volume overload decreased cardiac ejection fraction but treatment with RIC reversed this decrease in the ejection fraction to normal levels (Figure 7A). In summary, the results suggest that RIC releases exosomes that contain the myokine, stimulates sympathetic and bone marrow, and all these together mitigates the CHF (Figure 7B; Azevedo et al., 2001; Marsee et al., 2010; Kalani et al., 2014, 2016; Vicencio et al., 2015).

## Discussion

During epigenetic memory, the CHF reduces the endogenous capacity to produce a beneficial cardioprotective agent, H_2_S (*via* declining the activities of CBS and CSE levels), and this results in an increase in the Hcy which eventually creates hyperhomocysteinemia (HHcy, a condition also created by hypermethylation). These alterations are responsible for causing muscle myopathy (Veeranki and Tyagi, 2015; Veeranki et al., 2015). RIC may decrease epigenetic methylation, thereby inducing genes that help generate H_2_S production by increasing the CBS of muscle and CSE enzyme levels. Also, a decrease in epigenetic methylation leads to the induction of genes that trigger cell proliferation causing the bone marrow stem cell (BMSC) mobilization to the site of injury (Azevedo et al., 2001; Marsee et al., 2010; Tyagi et al., 2021). Interestingly, the intact skeletal muscle endocrine functions have been shown to be cardioprotective yet their relevance has not yet been tested in the postischemic recovery during CHF. This study underscores the significance of muscle endocrine function toward the recovery of the heart, and thus, its function meticulously verifies and validates our hypothesis. H_2_S-induced cardio-protective epigenetic modifications have not been studied in the CHF condition.

Accordingly, we utilized a multi-blot protein expression analysis to comprehensively evaluate “proteome to function” changes that appear to regulate CHF tolerance, regeneration, and recovery with and without RIC. Whether RIC enhances regeneration or engraftment survival after CHF demands urgent attention. Our study does suggest RIC influence on the bone marrow-derived mesenchymal stem cell survival, contribution toward revitalizing new myocardium, and its vasculature during CHF (Azevedo et al., 2001). The rationale is that RIC reverses the HFrEF-induced adverse environment in the myocardium to influence the survival of the homing BMSC. Many studies have reported the beneficial effects of RIC in the ischemic heart, due to the unpredictable nature of heart ischemia occurrence, especially acute myocardial infarction cases. While there have been few reports with regards to bone marrow supplementation, almost none have evaluated the long-term benefits of RIC on cardiac recovery during CHF (Azevedo et al., 2001). Therefore, this study proposes the long-term effects of RIC on CHF and the reduced ejection fraction of a failing myocardium. *Via* this study, the myocardial expression of the somatotropic axis, adrenergic signaling, and calcium handling genes during HF with preserved ejection fraction and HF with reduced ejection fraction have been successfully demonstrated, thus, emphasizing the reciprocal organ interactions during HF, a position paper from the ESC working group on myocardial function, and highlighting the importance of surgical ablation of the right greater splanchnic nerve for the treatment of HF with preserved ejection fraction (Ciccarelli et al., 2021; D’Assante et al., 2021; Málek et al., 2021). Previous study has shown that cardiomyocytes derived EVs injected into the hearts have been shown to promote cardiac recovery, and in this study, we showed that exosome released from the skeletal muscle contains musclin harboring exosomes that can protect the heart against the HF phenotype such as the CHF. In fact, the beneficial effects arising from EVs or exosomes have been found to have antiapoptotic, anti-fibrotic, and pro-angiogenic effects, all of which are crucial to restore the function of the damaged myocardium (Taylor et al., 1998; Miyagawa et al., 2010; Di Lascio et al., 2012).

## New and Noteworthy

During acute heart failure (HF), remote ischemic conditioning (RIC) is beneficial, but how it extends its benefits is unclear. Studies from our laboratory have shown three phases describing cardiopulmonary heart failure (CHF) viz. (1) HF with preserved ejection fraction (HFpEF), (2) HF with reduced EF (HFrEF), and (3) HF with reversed EF. Since reciprocal organ interaction, ablation of sympathetic, and calcium signaling genes are associated with HFpEF to HFrEF, the mechanism remains at large. We opined that a change in phenotype from compensatory HFpEF to decompensatory HFrEF is determined by a decrease in regenerative and proangiogenic factors along with an increase in epigenetic memory and inflammation. To prove this, we created CHF by aorta-vena-cava (AV) fistula in mice that were subsequently treated with that of hind-limb RIC. HFpEF vs. HFrEF transition was determined by serial/longitudinal echo parameters. Findings revealed an increase in skeletal muscle musclin contents, CD71, and sympathetic activation by RIC. We also noticed a decrease in vascular density and attenuation of endocardial endothelial – blood-heart barrier function due to an increase in MMP-2, VEGF, caspase, and calpain, all these alterations were mitigated by RIC-released skeletal muscle exosomes that contain musclin, the myokine along with bone marrow and sympathetic activation.

## Data Availability Statement

The raw data supporting the conclusions of this article will be made available by the authors, without undue reservation.

## Ethics Statement

The animal study was reviewed and approved by IACUC approved by University of Louisville School of Medicine, Louisville, KY, United States.

## Author Contributions

MS and ST conceived the research plan, designed the experiments, helped to analyze the data, wrote and edited the initial draft, and then finalized the manuscript. RH, YZ, and IS performed the experiments and helped to write the section “Materials and Methods” and figure legends in the initial draft of the manuscript. All authors read and approved the final version of this manuscript.

## Conflict of Interest

The authors declare that the research was conducted in the absence of any commercial or financial relationships that could be construed as a potential conflict of interest.

## Publisher’s Note

All claims expressed in this article are solely those of the authors and do not necessarily represent those of their affiliated organizations, or those of the publisher, the editors and the reviewers. Any product that may be evaluated in this article, or claim that may be made by its manufacturer, is not guaranteed or endorsed by the publisher.

## Abbreviations

DAAM: disheveled associated activator of morphogenesis
DNMT: DNA methyl transferase
FTO: fat-mass obesity-associated protein
Hcy: homocysteine
Met: methionine
mTORC1: mechanistic target of rapamycin complex 1
NLRP3: nod-like receptor family pyrin domain containing 3
PC: phosphatidylcholine
PCP: phosphatidylcholine phosphatase
PE: phosphatidylethanolamine
PEMT: phosphatidylethanolamine methyltransferase
REDD 1: regulated in development DNA damage response 1
SAHH: S-adenosine homocysteine hydrolase
SAM: S-adenosine methionine
SAH: S-adenosine homocysteine
TLR4: toll-like receptor-4.
